# Development and Validation of a Pyroptosis-Related Long Non-coding RNA Signature for Hepatocellular Carcinoma

**DOI:** 10.3389/fcell.2021.713925

**Published:** 2021-11-15

**Authors:** Zeng-Hong Wu, Zi-Wei Li, Dong-Liang Yang, Jia Liu

**Affiliations:** Department of Infectious Diseases, Union Hospital, Tongji Medical College, Huazhong University of Science and Technology, Wuhan, China

**Keywords:** hepatocellular carcinoma, pyroptosis, lncRNAs, TCGA, prognosis

## Abstract

**Background:** Hepatocellular carcinoma (HCC) is a highly aggressive malignant disease, and numerous studies have demonstrated that an inflammatory environment can induce normal cells to transform into cancerous.

**Methods:** We integrated genomic data to comprehensively assess the association between pyroptosis and tumor microenvironment (TME) cell-infiltrating characteristics in HCC, as well as the potential molecular function and clinical significance of lncRNA.

**Results:** The analysis of CNV alteration frequency displayed that CNV changes were common in 33 PRGs, and most were focused on copy number amplification. As a result of lasso regression analysis, nine differentially expressed lncRNAs (AL031985.3, NRAV, OSMR-AS1, AC073611.1, MKLN1-AS, AL137186.2, AL049840.4, MIR4435-2HG, and AL118511.1) were selected as independent prognosis factors of HCC patients. Patients at high risk have poorer survival than those in the low-risk group in training and testing cohorts. A low-risk score was significantly associated with an IC50 of chemotherapeutics such as bortezomib (*p* < 0.001), but a high-risk score was significantly linked to docetaxel (*p* < 0.001), implying that signature served as a prospective predictor for chemosensitivity.

**Conclusion:** This work suggests pyroptosis-related lncRNAs features and their potential mechanisms on tumor microenvironment. The exploration may assist in identifying novel biomarkers and assist patients in predicting their prognosis, clinical diagnosis, and management.

## Background

Hepatocellular carcinoma (HCC) is a highly aggressive malignant disease and is the third most common cause of cancer death worldwide (Bray et al., 2018). The most general risk factors for HCC are viral hepatitis infection, aflatoxin exposure, and non-alcoholic steatohepatitis (Coskun, 2017; Llovet et al., 2018). The early manifestations of HCC are not obvious, and its specificity is poor. As a result, early HCC is difficult to diagnose, and the 5-year overall survival (OS) rate is just 34–50% (Lang et al., 2007). Although early HCC can be curable by resection, liver transplantation, or ablation, most patients suffer from an unresectable disease, and there remain no beneficial complete curable ways for HCC patients (Hu et al., 2021). Many studies have revealed that molecular pathological subtypes and driver gene mutations significantly affect the prognosis of HCC patients. However, the underlying molecular pathologic mechanism of HCC development is complex, comprising diverse functions and multiple interactions between numerous genes. Accordingly, identifying the pathogenesis and regulatory network of HCC is urgent in determining therapeutic targets and screening the mechanism of targeted drugs.

Pyroptosis, also known as cell inflammatory necrosis, is a kind of programed cell death characterized by continuous expansion until the cell membrane ruptures, releasing the cell contents and activating an intense inflammatory response (Rühl et al., 2018). Numerous investigations have demonstrated that an inflammatory environment can transform normal cells into cancerous ones. While the inflammatory response environment formed by pyroptosis is conducive to tumor growth, it can also promote tumor cell apoptosis (Block et al., 2018). The body can recruit and activate caspase-1 protein through classical inflammasome pathways (canonical inflammasome pathways) or activate caspase 4/5/11 protein through the non-canonical inflammasome pathway to directly cleave and activate gasdermin protein D (*GSDMD*), which eventually result in membrane porosity and cell death (Jorgensen et al., 2017). There are accumulating evidences that pyroptosis-mediated cell survival participates in the etiology and progression of HCC. Long non-coding RNAs (lncRNAs) are RNA molecules whose transcriptional length exceeds 200 nt and regulates gene expression levels. Due to their involvement in various biological regulatory processes, lncRNAs are intimately associated with occurrence, development, and metastasis of tumors (Gupta et al., 2010). A recent study demonstrated that high expression of lncRNA SNHG7 inhibited NLR family domain, containing three (NLRP3) dependent pyroptosis in HepG2 and SK-Hep-1 cells, whereas knockdown of SNHG7 promoted expression levels of NLRP3, caspase-1, and interleukin 1β, causing pyroptosis (Chen et al., 2020). Additionally, studies have demonstrated that pyroptosis is inhibited in HCC tissues and cells, which may be a new tumor target for HCC treatment (Chu et al., 2016). Caspase-3 can shear *GSDME* following chemotherapy treatment, causing cell membrane perforation, thereby inducing cell pyroptosis. However, until now, very few studies have systematically evaluated the role of pyroptosis-related lncRNAs in HCC patients. This study integrated genomic data to thoroughly assess the link between pyroptosis and tumor microenvironment (TME) cell-infiltrating characteristics in HCC, as well as the potential molecular function and clinical significance of lncRNA and offer possible beneficial biomarkers for HCC prognosis.

## Materials and Methods

### Data Gathering

RNA-sequence data from 424 (50 normal and 374 tumors, FPKM value) samples, somatic mutation data, and copy number variation (CNV) were collected from The Cancer Genome Atlas--Hepatocellular Carcinoma (TCGA-HCC,^1^ date up to 10 April 2021) database. The FPKM value is converted to transcripts per kilobase million (TPM) values. The batch effect from non-biotech bias is corrected through the “combat” algorithm based on the SVA R package. We extracted 33 pyroptosis-related genes (PRGs) from the study of Ye et al. (2021) (Supplementary Table 1). GTF files were obtained from Ensembl for annotation to identify lncRNAs and mRNAs. The connection was considered significant if the correlation coefficient | *R*2| > 0.3, and *p* < 0.001 between mRNAs and lncRNAs. Differential expression of lncRNAs or mRNAs was decided at FDR < 0.05 and | log2FC| ≥ 1 using the R software, limma package. A heuristic strategy is employed to determine the 33 PRGs that are epigenetically activated (EA) or epigenetically silenced (ES) in tumors compared with their DNA methylation status in normal tissues in different cancer types (Wang et al., 2018). This way prioritized the mRNAs with significant differences in DNA methylation and suggested expression changes that were highly correlated with their DNA methylation alterations.

### Unsupervised Clustering for lncRNAs

Based on their expression of prognosis-related lncRNAs, unsupervised cluster analysis was performed to identify different pyroptosis modification patterns, and patients were classified for further analysis. The consensus clustering algorithm determines the number and stability of clustering. The ConsenSuClusterPlus R package was used to process the above analysis, and 1,000 repeats were performed to ensure the stability of the classification. To determine the differences in biological processes between pyroptosis modification models, we performed Gene Set Variation Analysis (GSVA) enrichment analysis based on the “GSVA” R package. The gene set of “c2.cp.kegg. V6.4. symbols” was obtained from the MSIGDB online tool for GSVA analysis. GSVA is a non-parametric, unsupervised method for estimating genome-enrichment variation from samples of expressed datasets.

### Building and Validating the Prognostic Pyroptosis-Related lncRNA Signature

The data partition is created using a functional R packet caret to divide HCC samples into training and testing sets. Univariate and multivariate Cox regression analyses were used to evaluate the prognostic value of pyroptosis-related lncRNAs. Meanwhile, the significant lncRNAs in univariate and multivariate Cox regression analyses were selected as characteristic lncRNAs. As a risk score, the prognostic signature = esum (lncRNA’s expression × coefficient). The risk score of individual patients was also counted. Based on the median risk score value, the set was divided into high- and low-risk groups, and ROC curves were utilized to predict the accuracy of prognostic signatures. The Kaplan–Meier curve was used to examine the impact of the pyroptosis-related lncRNA signature on patient survival.

### Function Analysis Between High- and Low-Risk Groups

Gene Set Enrichment Analysis (GSEA) is employed to evaluate the contribution of genes to a phenotype by analyzing their distribution trend in a predefined gene set in the gene table ranked by phenotype relevance (Zhang et al., 2020). GSEA was used to investigate the potential biological function of lncRNA. To assess the signature in clinical trials for HCC treatment, we utilized R ggplot2 and pRRophetic packages to calculate the lower half inhibitory concentration (IC50) of commonly used chemotherapeutic drugs (such as cisplatin) in TCGA-HCC. The method works by building statistical models from gene expression and drug sensitivity data in a very large panel of cancer cell lines and then applying these models to gene expression data from primary tumor biopsies (Geeleher et al., 2014). Moreover, somatic mutations were explored among high- and low-risk groups using maftools, an R package for analyzing and visualizing mutation annotation format (MAF) files from large-scale sequencing studies (Xu et al., 2021). Tumor mutation burden (TMB) is defined as the total number of somatic gene-coding errors, base substitutions, gene insertion, or deletion errors detected per million bases. TMB has been depicted as an indicator of immunological reaction and tumor behavior (Birkbak et al., 2013; Goodman et al., 2017). Mutation formation advances carcinogenesis by activating or inactivating genes and related pathways, generating novel peptide sequences that can animate immune reaction (Klebanov et al., 2019). Thus, we explored the connection between the high/low score group and TMB.

### Immunity and the Risk Groups

The lollipop of immune responses is based on XCELL (which uses single-sample gene set enrichment analysis to estimate the abundance scores of 64 immune cell types), TIMER (which uses RNA-seq expression profile data to detect immune cell infiltration in tumor tissues), QUANTISEQ (based on deconvolution algorithm, RNA-seq data of bulk samples are used), MCPCOUNTER (which uses gene expression to estimate the population abundance of tissue-infiltrating immune and stromal cell populations), EPIC (uses RNA-seq expression profile data), CIBERSORT (a deconvolution algorithm using support vector regression for determining immune cell type in tumors), and CIBERSORT-ABS (utilizes deconvolution of mass gene expression information and a sophisticated algorithm for quantification of the cellular element of immune reaction) algorithms to analyze the Spearman correlation between risk score values and tumor-infiltrating immune cells (TIIC). In addition, the heatmap depicts the component differences of immune cells between the high- and low-risk groups, as well as risk score values.

### Statistical Analysis

The normally and non-normally distributed variables were analyzed using the unpaired Student’s *t*-test and Wilcoxon test, respectively. The signature and clinicopathological connection were assessed using the chi-square test. Logistic regression analyses were employed to determine whether the signature can be used as an independent clinical prognostic predictor. The CNV landscape of 33 PRGs in 23 pairs of chromosomes was analyzed using the R package of RCircos. The waterfall function of maftools R package was used to analyze mutations in patients with high- and low-risk score subtypes in TCGA-HCC cohort. For each analysis, the statistically significant setting was *p* < 0.05.

## Results

### Landscape of Genetic Variation of 33 Pyroptosis-Related Genes

**FIGURE 1:**
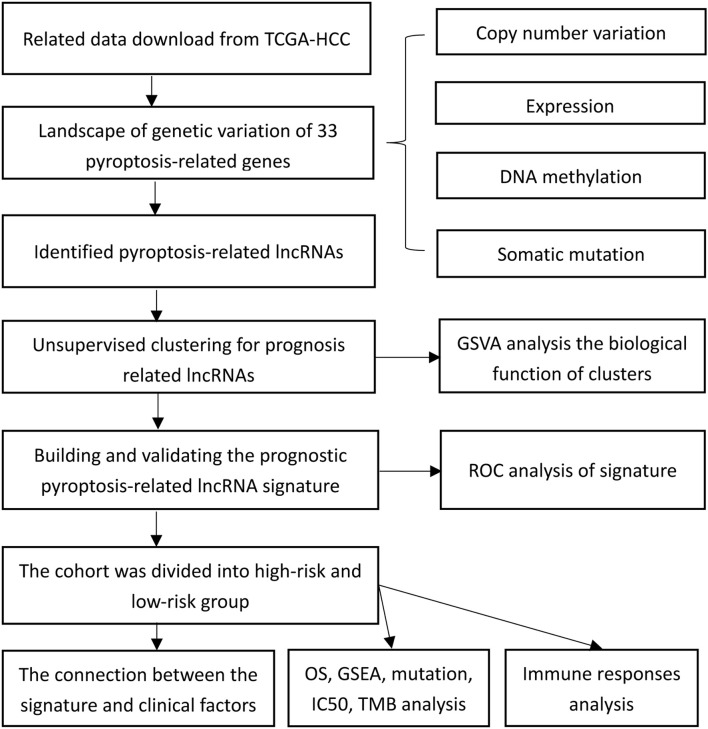
The flow chart showing the scheme of the study.

**FIGURE 2:**
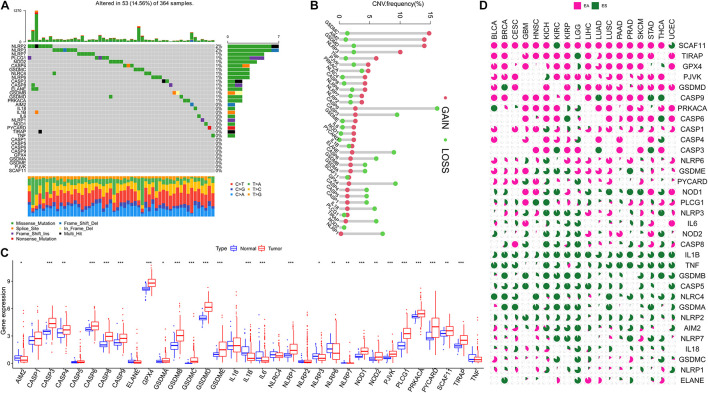
Landscape of 33 pyroptosis-related genes (PRGs) in hepatocellular carcinoma. **(A)** The occurrence of copy number variation (CNV) and somatic mutations of 33 PRGs in The Cancer Genome Atlas—Hepatocellular Carcinoma (TCGA-HCC): among 364 samples, 53 experienced mutations with a frequency of 14.56%, and *NLRP2/3* demonstrated the highest mutation frequency. **(B)** The analysis of CNV alteration frequency displayed that CNV changes were common in 33 PRGs; the result indicated that *GSDMC*, *AIM2*, and *GSDMD* had a general frequency of CNV gain, and *CASP9* had a widespread frequency of CNV loss. **(C)** The expression of 33 PRGs between normal and tumor cells in TCGA-HCC. **(D)** The impact of 33 PRGs on EA or ES in different cancer types; the result suggested that *CASP* family genes mainly affect EA, whereas the *NLRP* family genes mainly affect ES; pink represents EA and green represents ES. EA, epigenetically activated; ES, epigenetically silenced. The asterisks represent the statistical *p*-value (*p < 0.05; **p < 0.01; ***p < 0.001).

Figure 1 presents a flow chart of this study scheme. The information of clinicopathological characteristics of 374 HCC patients is shown in Table 1. The occurrence of CNV and somatic mutations of 33 PRGs in TCGA-HCC is summarized in Figure 2A. Among 364 samples, 53 experienced mutations with a frequency of 14.56%, and *NLRP2/3* demonstrated the highest mutation frequency, and most samples had nonsense mutation. The analysis of CNV alteration frequency revealed that CNV changes were common in 33 PRGs, and most were focused on copy number amplification, while *GSDMC, AIM2*, and *GSDMD* had a general frequency of CNV gain, and *CASP9* had a widespread frequency of CNV loss (Figure 2B). Meanwhile, the location of CNV alteration of these genes on chromosomes is displayed in Supplementary Table 2. The expression of these genes between normal and tumor tissues in TCGA-HCC is illustrated in Figure 2C. We also determine whether PRG expression is regulated by DNA methylation changes at their promoters. The impact of 33 PRGs on EA and ES in different cancer types is depicted in Figure 2D. The result suggested that *CASP* family genes mainly affect EA, whereas the *NLRP* family genes primarily influence ES. In general, PRGs with amplified CNV were significantly highly expressed in tumor tissues and *vice versa*. Additionally, we observed that some non-mutated genes accompanied high expression and were associated with DNA methylation. The above analysis revealed a high level of heterogeneity in genetic and expression patterns of pyroptosis in HCC, indicating that pyroptosis plays a critical role in tumor occurrence and development.

**TABLE 1:** The information of clinicopathological characteristics of 374 hepatocellular carcinoma (HCC) patients.

**Clinical characteristics**		**Total (377)**	**%**
Age at diagnosis (y)		53 (16–90)	
Futime (m)		28.0 (0–122.5)	
Gender	Female/Male	122/255	32.4/67.6
Stage	I/II/III/IV/NA	175/87/86/5/24	46.4/23.1/22.8/1.3/6.4
Grade	G1/G2/G3/G4/NA	55/180/124/13/5	14.6/47.7/32.9/3.4/1.3
T-classification	T1/T2/T3/T4/TX/NA	185/95/81/13/1/2	49.1/25.2/21.5/3.4/0.3/0.5
M- classification	M0/M1/MX	272/4/102	72.1/1.1/27.1
N- classification	N0/N1/NX/NA	257/4/115/1	68.2/1.1/30.5/0.3
Status	Alive/Death	129/248	34.2/65.8

*Data express as Mean (min-max). NA: not applicable.*

### Cluster and Biological Function

**FIGURE 3:**
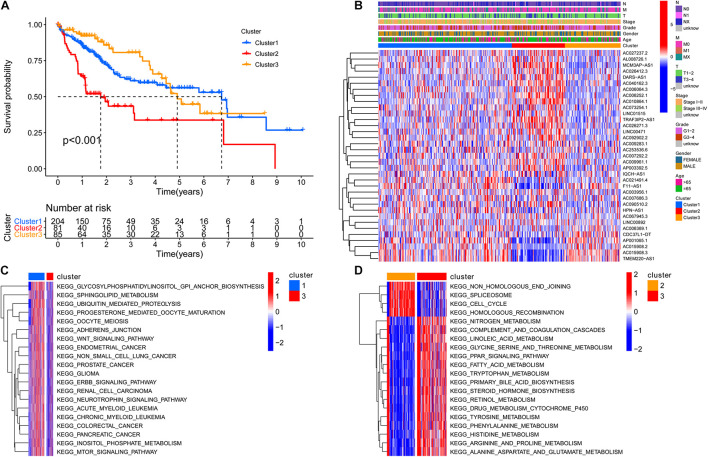
The cluster and biological function based on the expression of 336 differentially expressed lncRNAs. **(A)** Three different clusters were identified by the unsupervised clustering method, and the cluster 2 model indicated an especially survival disadvantage. **(B)** The heatmap of the top 20 long non-coding RNAs (lncRNAs) associated with favorable and poor overall survival (OS) and clinicopathological manifestations; the result shows that the expression of lncRNA in cluster 2 is obviously heterogeneous. **(C)** Cluster 1 was prominently correlated to cancer development, such as glioma and small-cell lung cancer. **(D)** Cluster 3 was mainly enriched in pathways related to metabolism activation such as fatty acid metabolism, beta alanine metabolism. N, lymph node; N0, the lymph nodes are not affected; N1, the lymph nodes are affected; NX, unevaluated; M, metastasis; M0, no metastasis; M1, metastasis; MX, unevaluated; T, primary tumor.

To screen for prognostic lncRNAs, differentially expressed lncRNAs were analyzed in univariate Cox analysis. Then, 336 lncRNAs that were important in univariate Cox analysis were included in multivariate Cox analysis (Supplementary Table 3). The ConsensusClusterPlus R package was applied to classify patients based on their expression of 336 lncRNAs. Finally, using unsupervised clustering method, three different clusters were identified, consisting of 204 cases of cluster-1, 81 cases of cluster-2, and 85 cases of cluster-3. Kaplan–Meier analysis suggested that the cluster-2 model was particularly detrimental to survival (Figure 3A). Following that, we examined the link between lncRNA expression and clinicopathological manifestations. The heatmap of the top 20 lncRNAs associated with favorable and poor OS and clinicopathological manifestations is demonstrated in Figure 3B. The results revealed that lncRNA expression in cluster 2 is obviously heterogeneous. GSVA enrichment analysis investigated the biological function between these different clusters. Cluster-1 was prominently correlated to cancer development such as glioma and small cell lung cancer (Figure 3C and Supplementary Table 4). Cluster 2 is mostly enriched for DNA damage repair processes such as non-homologous end joining, spliceosome, cell cycle, and homologous recombination. Cluster 3 was mainly enriched in metabolic activation pathways such as fatty acid metabolism and beta-alanine metabolism (Figure 3D and Supplementary Table 5). As a characteristic of pyroptosis, inflammasome is a multiprotein complex of molecular signals related to cell damage that can be activated by damaged double-stranded DNA, causing the production of inflammatory factors and GSDMD-mediated pyroptosis. As a result, we speculated that DNA damage repair in cluster 2 may activate pyroptosis and other cell cycle-related processes, resulting in a poor prognosis.

### Prognostic Pyroptosis-Related Long Non-coding RNAs in Hepatocellular Carcinoma

**FIGURE 4:**
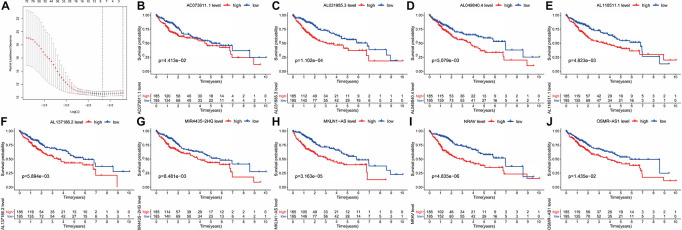
Kaplan–Meier curve analysis of the prognostic pyroptosis-related lncRNAs in TCGA-HCC. **(A)** Nine differently expressed lncRNAs were selected as independent prognosis factors based on lasso regression. **(B–J)** The cohort was divided into two groups (high and low risk) based on the median risk score, which was used as the cutoff value, and the high expression of the nine lncRNAs indicated a bad prognosis.

**FIGURE 5:**
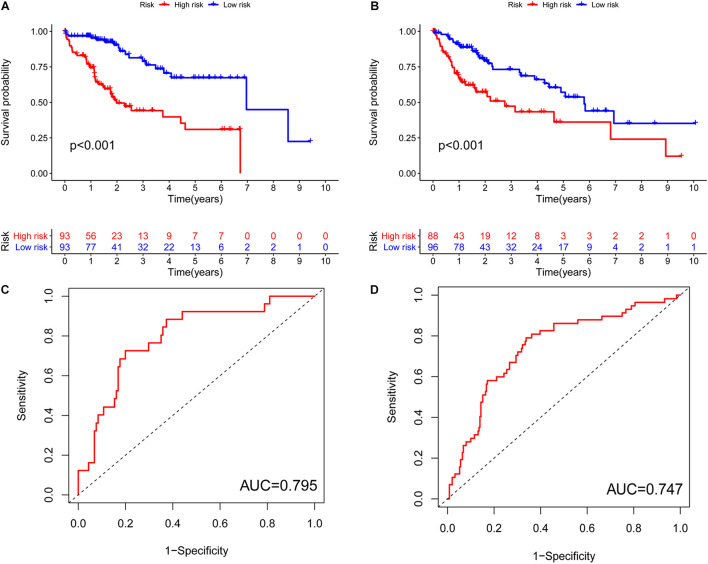
Characteristics of the pyroptosis-related lncRNA signature in the training set and testing cohort. **(A)** Kaplan–Meier analysis indicated that high-risk patients had poorer survival compared with the low-risk group in the training cohort. **(B)** Kaplan–Meier analysis indicated that high-risk patients had poorer survival compared with the low-risk group in the testing cohort. **(C)** ROC curves to determine whether the expression pattern of lncRNAs related to survival can be used as an early predictor of HCC and an AUC of 0.795 in the training set. **(D)** An AUC 0.747 in the testing set. AUC, area under curve.

**FIGURE 6:**
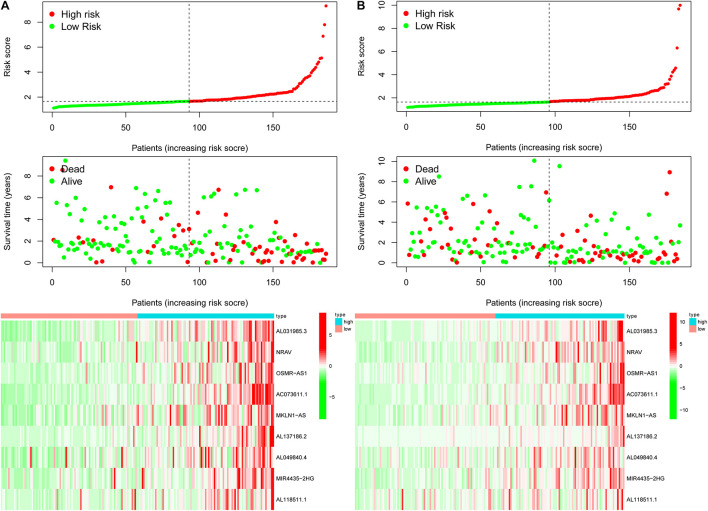
The risk survival status chart of TCGA-HCC cohort. **(A)** The risk survival status plot of the patient in the training cohort, and the number of patients who died increased with the increase in patient risk score. **(B)** The risk survival status plot of the patient in the testing set.

**FIGURE 7:**
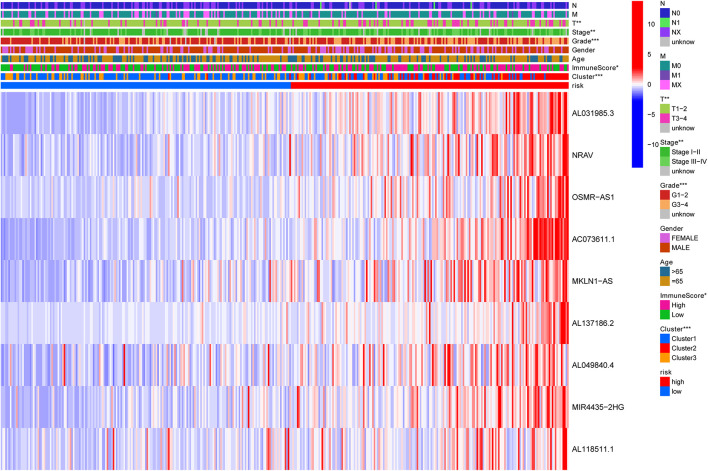
Heatmap to show the connection between clinical pathology, clusters, and risk scores. The results indicated that the expression of the nine lncRNAs in the high-risk group was significantly higher than that in the low-risk group, and this trend was more obvious in cluster 2. N, lymph node; M, metastasis; T, tumor.

Previously, univariate Cox analysis (using R package “survival” cox.ph and cox.zph functions) identified 336 lncRNAs that were significantly associated with survival, and then, the correlation coefficient of each lncRNA in the model was calculated to determine the possible colinearity between lncRNAs, and then a second regression based on these lncRNAs was performed to simultaneously detect whether multiple lncRNAs are related to survival. As a result, nine differentially expressed lncRNAs (AL031985.3, NRAV, OSMR-AS1, AC073611.1, MKLN1-AS, AL137186.2, AL049840.4, MIR4435-2HG, and AL118511.1) were selected as an independent prognostic factor based on the lasso regression in HCC (Figure 4A and Supplementary Table 6). Moreover, we calculated the risk score for each patient. The cohort was divided into two groups (high and low risk), and the median risk score was used as a cutoff value for training (186 cases) and testing (184 cases) sets. Thus, our model has the following formula: risk score = (0.189 × expressionAL031985.3) + (0.012 × expression NRAV) + (0.205 × expressionOSMR-AS1) + (0.244 × expressionAC073611.1) + (0.182 × expressionMKLN1-AS) + (0.226 × expressionAL137186.2) + (0.004 × expression AL049840.4) + (0.005 × expressionMIR4435-2HG) + (0.024 × expressionAL118511.1). Meanwhile, the high expression of these nine lncRNAs was associated with a poor prognosis (Figures 4B–J). Kaplan–Meier curves of the two cohorts can investigate the predicted value of signature based on nine lncRNAs found in OS. In the training and testing cohorts, high-risk patients had poorer survival than the low-risk group (Figures 5A,B). Additionally, we employed ROC curves to determine whether the expression pattern of lncRNAs related to survival can be used as an early predictor of HCC. Here, we found that the AUC of the training and testing sets was 0.795 and 0.747, respectively (Figures 5C,D). Additionally, we established a risk survival status chart of patients, and the number of patients who died increased as patient risk score increased (Figures 6A,B). Following that, to establish a prognostic model based on nine lncRNAs, we utilized univariate and multivariate Cox analyses to identify risk factors. Finally, signatures based on nine lncRNAs were established as independent prognostic factors (Table 2). Finally, the connection between clinical pathology, clusters, and risk scores was also explored, and the results demonstrated that the risk score was significantly related to tumor grade, stage, T stage, and clusters (Supplementary Figure 1). The heatmap results indicated that the high-risk group had significantly higher expression of nine lncRNAs than the low-risk group, and this trend was more obvious in cluster 2 (Figure 7). In addition, the impacts of risk scores on patient OS in different clinical subtypes were explored, and the results indicated that tumor grade, tumor stage, and T stage subtypes were significantly correlated with the survival of a patient (Supplementary Figure 2).

**TABLE 2:** The univariate and multivariate Cox analysis to determine the independent prognostic factors.

	**Univariate**		**Multivariate**	

**Item**	**HR (HR.95L-HR.95H)**	***P-*value**	**HR (HR.95L-HR.95H)**	***P-*value**
Training set				
Age	1.015 (0.993–1.038)	0.184	1.010 (0.987–1.033)	0.417
Gender	0.623 (0.358–1.085)	0.095	0.714 (0.396–1.288)	0.263
Grade	1.207 (0.800–1.822)	0.370	1.326 (0.832–2.115)	0.235
Stage	1.554 (1.156–2.088)	0.003	0.749 (0.298–1.885)	0.540
T	1.543 (1.168–2.040)	0.002	1.814 (0.740–4.450)	0.193
M	1.782 (0.968–3.281)	0.064	1.893 (0.891–4.022)	0.097
N	1.179 (0.587–2.368)	0.643	0.907 (0.380–2.168)	0.827
Risk score	1.623 (1.404–1.876)	<0.001	1.594 (1.359–1.868)	<0.001
Testing set				
Age	1.005 (0.985–1.025)	0.620	1.010 (0.989–1.031)	0.351
Gender	1.119 (0.644–1.942)	0.691	1.252 (0.707–2.219)	0.441
Grade	1.113 (0.799–1.551)	0.528	1.264 (0.882–1.811)	0.203
Stage	1.764 (1.310–2.375)	<0.001	1.603 (0.580–4.436)	0.363
T	1.711 (1.291–2.269)	<0.001	1.175 (0.440–3.138)	0.747
M	1.204 (0.667–2.175)	0.538	1.504 (0.752–3.007)	0.248
N	1.183 (0.681–2.057)	0.551	0.944 (0.486–1.834)	0.865
Risk score	1.010 (0.918–1.113)	0.032	1.014 (0.903–1.138)	0.018

### Gene Set Enrichment Analysis

**FIGURE 8:**
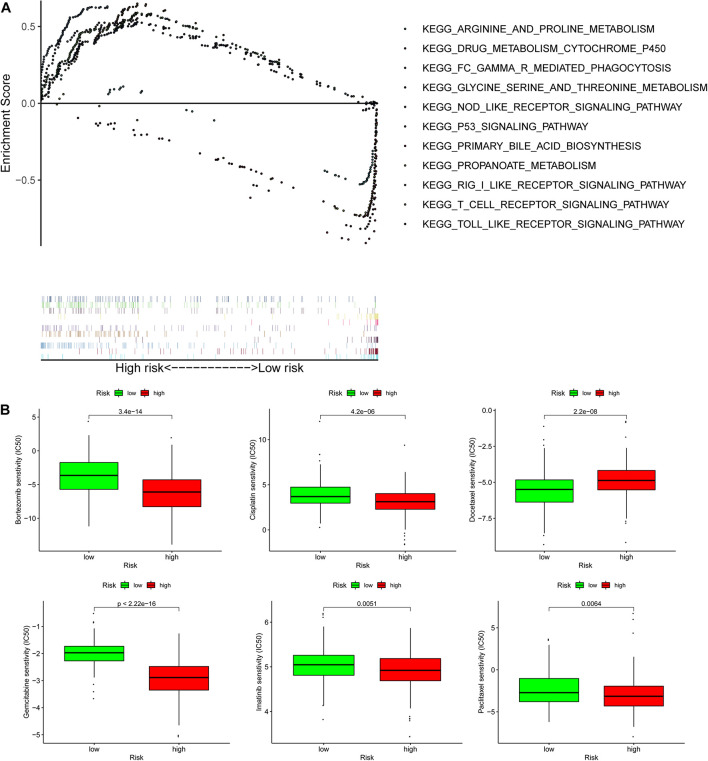
Gene set enrichment analysis (GSEA) and lower half inhibitory centration (IC50) of chemotherapeutic drugs between the high- and low-risk group based on the pyroptosis-related lncRNA signature in TCGA-HCC. **(A)** GSEA results suggested that the two risk groups were mainly enriched in the tumor-, immune-, and metabolism-related pathways. **(B)** A low-risk score was related to an IC50 of chemotherapeutics such as bortezomib, and a high-risk score was related to docetaxel.

**FIGURE 9:**
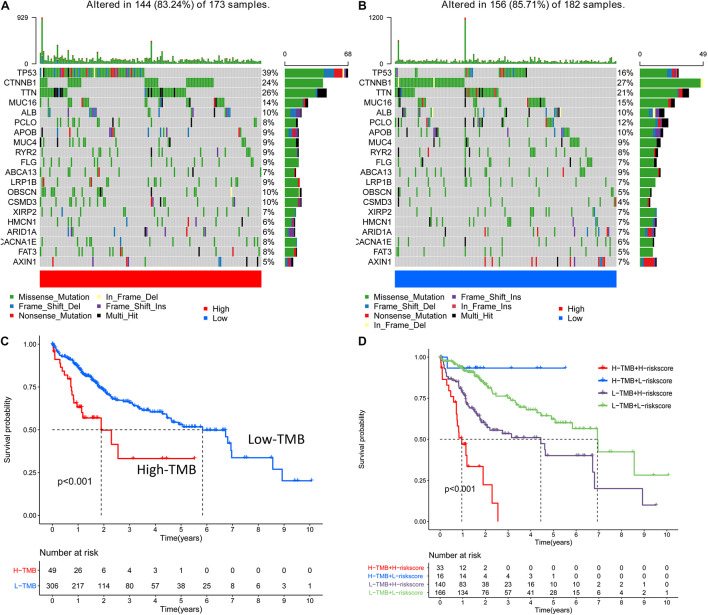
The result of alteration frequency and tumor mutation burden (TMB) between the high- and low-risk group. **(A)** The top 20 driver genes with the highest alteration in the high-risk group. **(B)** The top 20 driver genes with the highest alteration in the low-risk group. **(C)** The connection among the high-/low-score group and TMB, and the results show that the high TMB group demonstrated a poorer prognosis. **(D)** When high/low TMB is randomly combined with high/low risk, and high TMB is combined with a high risk score indicated a poorer prognosis.

**FIGURE 10:**
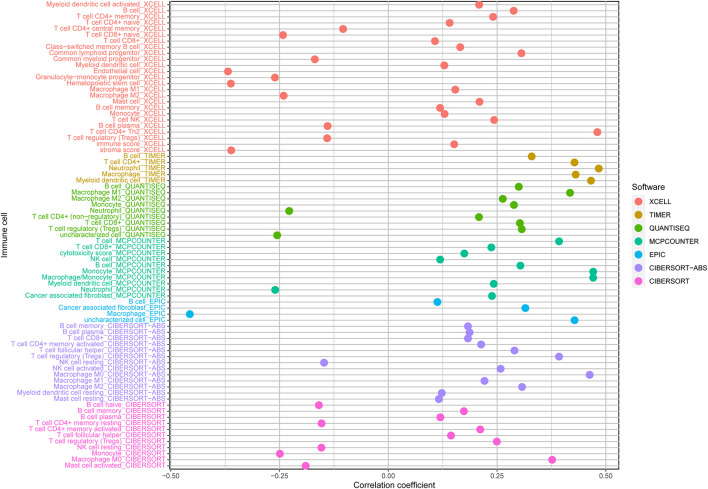
A detailed Spearman correlation analysis was also performed using different algorithms; a lollipop shape to exhibited the result, and from the results, we can find that most immune cells have a positive correlation with the risk score. TIMER, tumor immune estimation resource.

**FIGURE 11:**
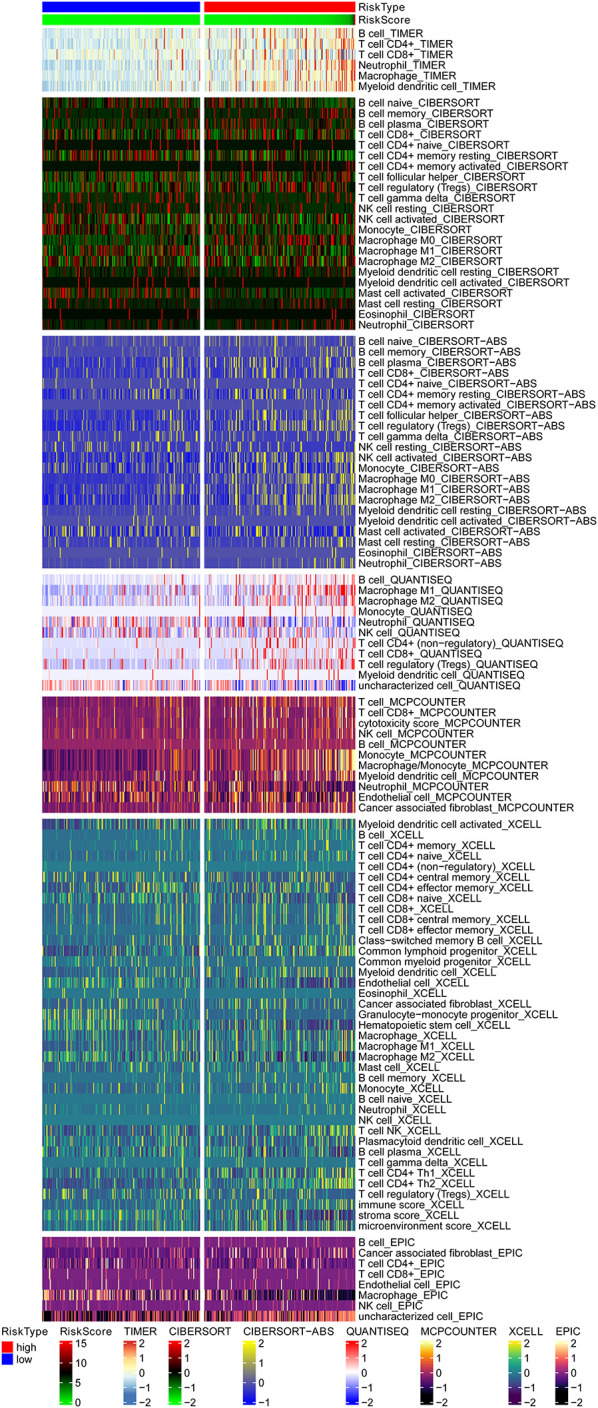
The heatmap of immune responses based on different algorithms among the high- and low-risk group, and the results demonstrated that most immune cells showed a trend of high expression in the high-risk group when compared with the low-risk group. TIMER, tumor immune estimation resource.

Biological functional annotation was performed through GSEA to identify potential enrichment pathways of risk groups. The results revealed that the two groups (high- vs. low-risk; high phenotypic enrichment) were mainly enriched in tumor- and immune-related pathways such as RIG-I-like receptor/P53/Toll-like signaling pathway and T-cell signaling pathway. While enrichment in phenotype was low, it was mainly concentrated on metabolism-related pathways, such as glycine serine and threonine metabolism, and propanoate metabolism (Figure 8A). Therefore, we speculate that our prognostic signature is related to tumor immunity and metabolism. Following that, we examined whether the risk score can predict the sensitivity of patients to chemotherapy and found that a low-risk score was linked to an IC50 of chemotherapeutics such as bortezomib (*p* < 0.001), whereas a high-risk score was linked to docetaxel (*p* < 0.001), implying that signature served as a prospective predictor for chemosensitivity (Figure 8B). Meanwhile, the top 20 driver genes with the highest alteration frequency of *TP53*, *CTNNB1*, *TTN*, and *MUC16* were significantly different between high- (Figure 9A) and low-risk groups (Figure 9B). TMB has been shown to play a critical role in tumor prognosis. As a result, we investigated the connection between high-/low-score group and TMB, and the results revealed that the high TMB group demonstrated a poorer prognosis (Figure 9C). Meanwhile, we found that a poorer prognosis was achieved when high TMB was combined with a high-risk score (Figure 9D). This implies that our signature is significantly correlated with TMB in HCC. Finally, we examined component differences of immune cells between high- and low-risk groups, as well as risk score values. A detailed Spearman correlation analysis was performed using different algorithms, with a resulting lollipop shape, as displayed in Figure 10. The results indicate that most immune cells are positively correlated with the risk score, consistent with our GSEA finding that the high-risk group is predominantly enriched in immune-related pathways. In addition, the heatmap of immune responses based on different algorithms is illustrated in Figure 11. The results demonstrated that most immune cells (T cell, B cell, and macrophages M1/M2) expressed at a higher level in the high-risk group than in the low-risk group. Tumor-associated macrophages are a new targeting strategy to boost anticancer therapy and a key player in TME regulation, where they have been demonstrated to promote tumor growth, angiogenesis, and metastasis (Abdin et al., 2021). As a result, our findings may provide new sights for deep macrophages in HCC. Finally, we analyzed *PD-L1* expression in the two risk groups and found that it was also significant in high- and low-risk groups (Supplementary Figure 3). In total, the signature may act for pyroptosis-related lncRNA status of HCC patients and present possible biomarkers for clinical therapeutic intervention.

## Discussion

Combining immunotherapy, radiotherapy, chemotherapy, and targeted therapy to suppress tumor progression via synergistic effect of multiple mechanisms may improve poor prognosis of tumor patients. Although the causes of tumors are more complicated, inflammatory reactions may be related factors. Long-term chronic inflammatory reactions can lead to local tissue dysplasia and, thus, carcinogenesis. Since the inflammasome is the key molecule that directs caspase-1 to pyroptosis, this could be a critical node in the relationship between tumor cells and pyroptosis. This study determined an effective pyroptosis-related lncRNA prognostic characterization and immune cell infiltration dysfunction in HCC patients. The investigation may assist in our understanding of the pyroptosis status and antitumor immune response in HCC and, thus, provide potential biomarkers for clinical therapeutic intervention.

Overall, our analyses uncovered nine differentially expressed lncRNAs (AL031985.3, NRAV, OSMR-AS1, AC073611.1, MKLN1-AS, AL137186.2, AL049840.4, MIR4435-2HG, and AL118511.1) as independent prognosis factors of HCC patients. AL031985.3 is both an autophagy and immune-related lncRNA and can be used in predicting HCC prognosis (Jia et al., 2020; Kong et al., 2020). NRAV was mainly located in the cytoplasm and was involved in regulating vesicle-transporting protein function (Li et al., 2020). Meanwhile, NRAV reduction is a portion of host antiviral innate immune response to virus infection through suppression of interferon-stimulated gene transcription (Ouyang et al., 2014). A study revealed that OSMR-AS1 was associated with aggressive phenotypes, unfavorable prognosis, and treatment resistance in glioma (Liang et al., 2020). MKLN1-AS was overexpressed and contributed to poor prognosis in HCC patients (Guo et al., 2021). In addition, MKLN1-AS increases *HDGF* expression by functioning as a molecular sponge for miR-654-3p, resulting in a cancer-promoting effect during the HCC process (Gao et al., 2020). MIR4435-2HG can promote immunometabolic activities of primary myeloid dendritic cells by targeted epigenetic modifications of a part of mTOR signaling pathway (Hartana et al., 2021). Similarly, MIR4435-2HG promotes proliferation and metastasis of HCC through modulating miR-22-3p/YWHAZ axis (Shen et al., 2020). In total, lncRNAs identified by our research may play a key role in carcinogenesis, as there has been no study about the role of AC073611.1, AL137186.2, AL049840.4, and AL118511.1 in cancers, and our study can provide useful information for future in-depth research.

Pyroptosis plays an antitumor role by activating immune response. Due to the defect in cell membrane, pyroptotic cells liberate a large amount of cellular substance, causing a strong inflammatory response and a widespread lymphocyte infiltration. Lymphocyte infiltration was significantly increased, which further induced caspase-3-independent and -dependent tumor cell pyroptosis, thus, forming positive feedback to enhance antitumor function (Wang et al., 2020). Additionally, a study indicated that lncRNAs could directly regulate pyroptosis on inflammasome. LncRNA Neat1 is associated with *NLRP3*, *NLRC4*, and *AIM2* inflammasomes in mouse macrophages to increase their assembly and subsequent pro-caspase-1 processing (Zhang et al., 2019). ROS-induced lysosome malfunction is a vital inducement of pyroptosis, and the arginine metabolism pathway participates in NLRP3 inflammasome inhibition (Jiang et al., 2021; Li et al., 2021). Glycolytic metabolism can impose stress on DNA replication and activate *AIM2* inflammasome (Fidler et al., 2021). In our study, cluster 3 was mainly enriched in pathways involved in metabolism activation, indicating the important role of metabolism in regulating pyroptosis function. Moreover, we found that most immune cells have a positive correlation with the risk score, implying the presence of significant differences in the compositions of immune cell types among risk groups.

As a newly discovered method of cell death, pyroptosis exhibits two consequences on tumors. On the one hand, key inflammatory bodies in pyroptosis can promote tumor cell death and inhibit proliferation and metastasis of tumor cells. On the other hand, the accumulation of inflammasomes is conducive to a microenvironment formation suitable for tumor cell growth, proliferation, and metastasis. In this study, several lncRNA biomarkers were integrated to determine their impact on patient outcomes. This could help identify novel biomarkers and precise therapeutic targets in HCC. In addition, this study may assist in prognostic prediction, diagnosis, and management strategies for HCC patients. However, further independent studies are required to confirm these results and lncRNAs associated with predictive apoptosis. Finally, this study has limitations. First, the results were not validated in clinical samples. Second, the results do not provide accurate clinical data due to the relatively small number of patients. Although this work investigates the prospect of developing a prognostic model, it still needs improvement.

## Conclusion

This work suggests pyroptosis-related lncRNA features and their potential mechanisms on tumor microenvironment. This research could help identify novel biomarkers and assist patients in predicting their prognosis, clinical diagnosis, and management.

## Data Availability Statement

The original contributions presented in the study are included in the article/Supplementary Material, further inquiries can be directed to the corresponding author/s.

## Author Contributions

Z-HW designed and analyzed the research study. Z-HW and JL wrote and revised the manuscript, and collected the data. JL was involved in the collection of new-added data, manuscript polishing (including the grammar modification, re-writing, and review), and the writing of the response to reviewers, gave the final approval of the version to be published and agreement to be accountable for all aspects of the work, and JL provided tumor tissues for the study. JL and D-LY also provided the financial support in professional English language editing and review services. All authors have read and approved the manuscript.

## Conflict of Interest

The authors declare that the research was conducted in the absence of any commercial or financial relationships that could be construed as a potential conflict of interest.

## Publisher’s Note

All claims expressed in this article are solely those of the authors and do not necessarily represent those of their affiliated organizations, or those of the publisher, the editors and the reviewers. Any product that may be evaluated in this article, or claim that may be made by its manufacturer, is not guaranteed or endorsed by the publisher.

## Footnotes

1
https://portal.gdc.cancer.gov/
