# Supplementary material for: Development and Validation of a Pyroptosis-Related Long Non-coding RNA Signature for Hepatocellular Carcinoma
Source: Front Cell Dev Biol. 2021 Nov 15;9:713925. doi: 10.3389/fcell.2021.713925 (PMC8634266; doi:10.3389/fcell.2021.713925)
Supplement: Supplementary file 1 [file Data_Sheet_1.docx]

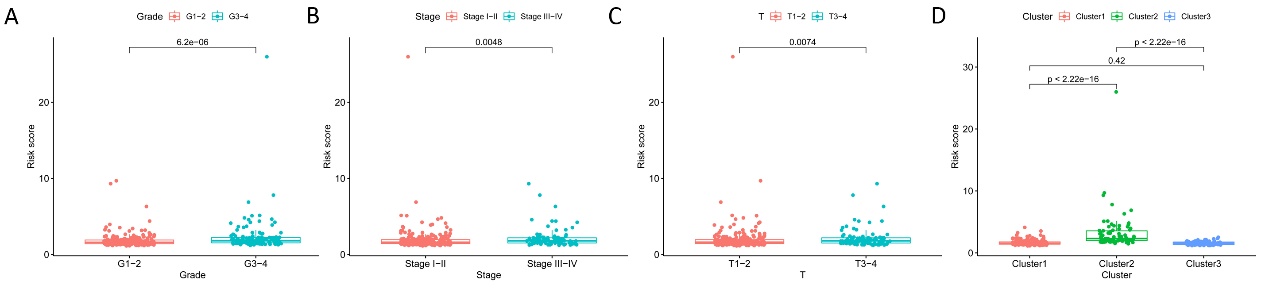


Figure S1: Scatter plot to shown the connection between clinical pathology, clusters and risk scores. A. The risk score was significant related to tumor grade; B. The risk score was significant related to tumor stage; C. The risk score was significant related to tumor T stage; D. The risk score was significant related to clusters (cluster 1 vs. cluster 2 and cluster 2 vs. cluster 3).


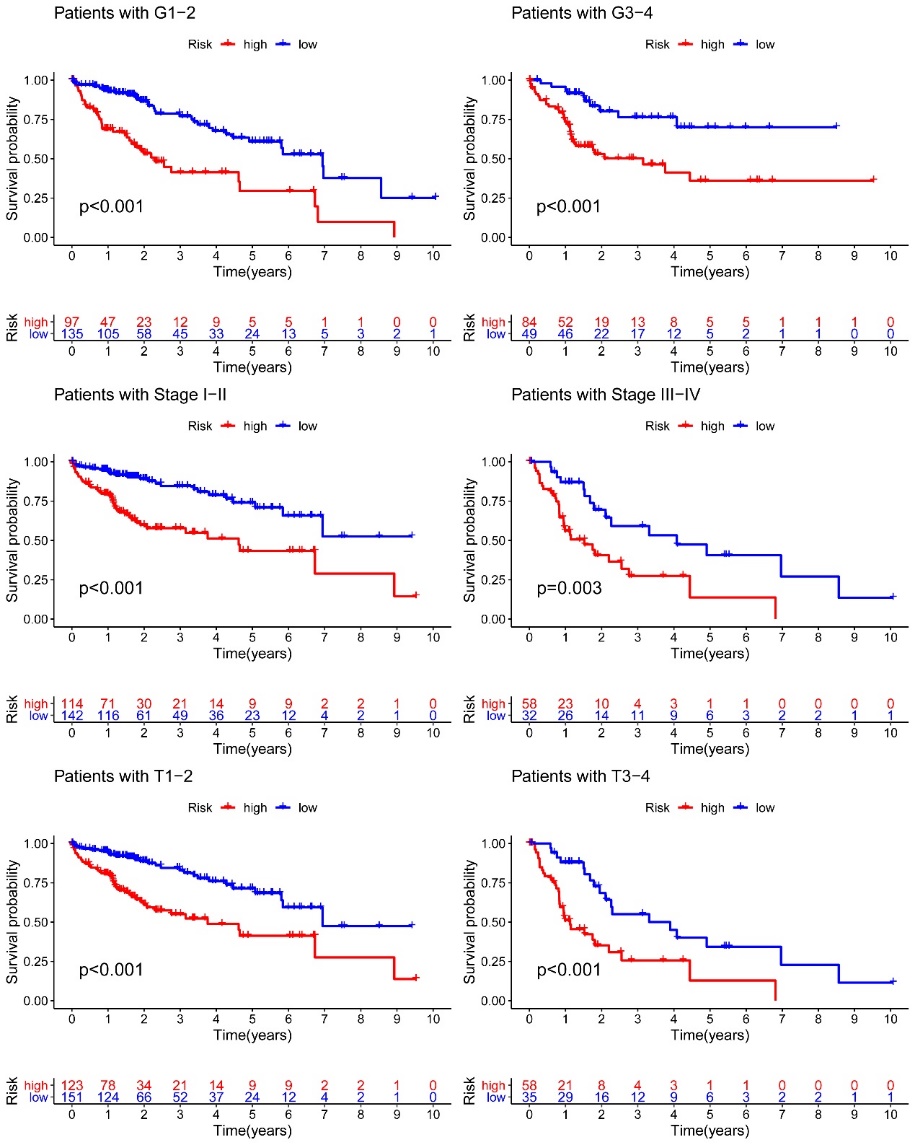


**Figure S2**. The impacts of risk scores on patient overall survival in different clinical subtypes and the results suggested that tumor grade, tumor stage and T stage subtypes significance related to patient’s survival.


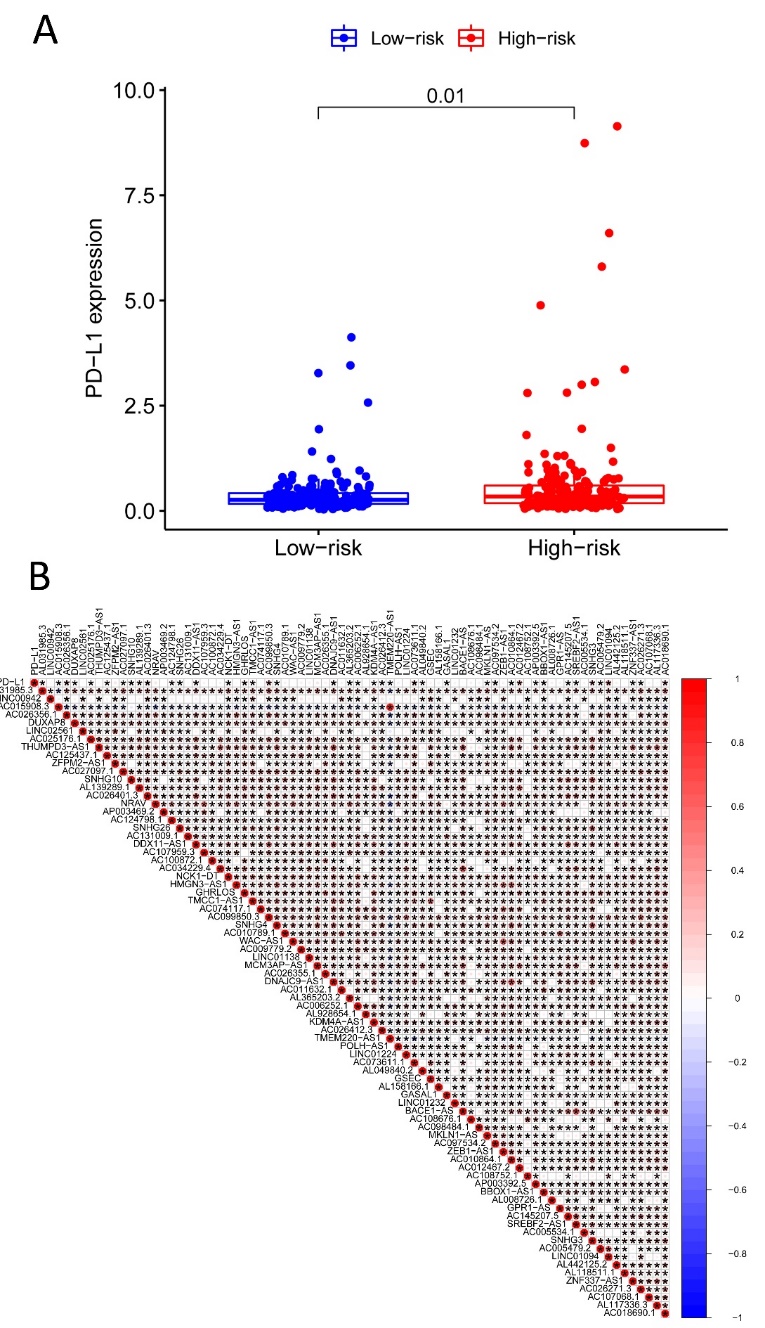


**Figure S3**. The relationship between *PD-L1* and the high and low risk group. A. The high expression of *PD-L1* in high risk group; B. The correlation between *PD-L1* and the prognosis-related lncRNAs in TCGA-HCC.

Supplement tables legends:

**Table S1**. The information of 33 pyroptosis-related genes (PRGs).

**Table S2**. The location of CNV alteration of pyroptosis-related genes on chromosomes.

**Table S3**. The univariate Cox analysis identified 336 pyroptosis-related lncRNAs.

**Table S4**. GSVA enrichment analysis investigate the biological function in cluster 1.

**Table S5**. GSVA enrichment analysis investigate the biological function in cluster 3.

**Table S6**. The differently expressed lncRNAs were selected as independent prognosis factors of HCC patients based on the lasso regression.
